# Experimental Studies of the Effect of *Schisandrachinensis* Extract on the State of Adaptive Capabilities of Rats under Chronic and General Exposure to Cold

**DOI:** 10.3390/ijerph182211780

**Published:** 2021-11-10

**Authors:** Irina Sergeeva, Tatyana Kiseleva, Valentina Pomozova, Nataliy Shkrabtak, Nina Frolova, Alexander Vereshchagin

**Affiliations:** 1 Department of Technology of Food Products from Plant Raw Materials, Kemerovo State University, 650000 Kemerovo, Russia; kisseleva.tf@mail.ru; 2 Scientific and Educational Center “Technologies for Innovative Development” Ural State Economic University, 620144 Yekaterinburg, Russia; pomozo.va@mail.ru; 3 Department of Life Safety, Amur State University, 675000 Blagoveshchensk, Russia; mmip2013@mail.ru (N.S.); ninelfr@mail.ru (N.F.); 4 Biysk Technological Institute (Branch), 659305 Biysk, Russia; val@bti.secna.ru

**Keywords:** *Schisandrachinensis* (Turcz.) Baill, cold, food, biologically active substances

## Abstract

Currently, there is an objective need to create fortified food products that allow not only to provide the body with energy, but also to replenish the deficiency of essential nutrients. A generalization of the information published by Rospotrebnadzor and the Institute of Nutrition of the Russian Academy of Medical Sciences indicates a deficiency in the diet of Russians of vitamins C, group B and β-carotene and minerals, including calcium and iron, regardless of the season of the year. The identified deviations lead to a violation of the immune status, a decrease in the body’s resistance to infections, and other unfavorable environmental factors, leading to an increase in the level of morbidity and a decrease in working capacity. The main unfavorable climatic factor that the population of the Far Eastern region has to face is low freezing temperatures. Adaptation to cold exposure is a complex process that requires a long period and may be accompanied by functional disorders and morphological changes in body tissues. In connection with the above, the problem of increasing the adaptive capabilities of a person to unfavorable environmental factors by means of correcting daily nutrition, providing the body with essential macro- and micronutrients, which is important in the prevention of possible diseases, is of particular importance. This study is aimed at assessing the effect of *Schisandrachinensis* extract on the adaptive capacity of rats in conditions of chronic and general cold. It was found that the extracts obtained from the fruits of *Schisandra chinensis* are characterized by a high content of biologically active substances. In experiments with determining the duration of running on the treadmill, a distinct act-protective effect was observed with the introduction of *Schisandra chinensis* extracts at a dose of 150 mg/day, against the background of reduced resistance to physical activity due to cold exposure. It was found that exposure to cold significantly reduced the swimming resistance of rats on all days of the study. The introduction of *Schisandra chinensis* extract into the diet led to an increase in resistance to fatigue and an increase in the duration of swimming on all days of the experiment. Conclusions: in this experimental model, a gradually increasing effect of increasing the physical performance of rats was demonstrated with prolonged (28 days) intake of the developed drinks, which coincides with the literature data on a number of other adaptogens and indicates the presence of cumulative properties of biologically active substances of Schisandra extract.

## 1. Introduction

*Schisandra chinensis* (Turcz.) Baill is a perennial deciduous woody vine. The fruit is a team, a juicy multileaf, formed due to the growth of carpels. The taste of the fruit pulp is extremely sour, the skin is sweet. The seeds consist of a hard, fragile peel and a dense core, the smell when rubbed is strong, specific, the taste is bitter-burning, spicy, and the whole berry is salty, therefore in Chinese medicine Schisandra is called the “berry of five tastes” [1,2,3,4].

Nutritional value, taste and aroma of fruits are determined by their chemical composition. From this point of view, the fruits can have a therapeutic or therapeutic and prophylactic effect on the human body.

Schisandra fruits contain 82 to 87% water. The high water content in the tissues determines the high activity of the biochemical processes occurring in them, the turgor state of the cells, their juiciness and freshness. First of all, Schisandra fruits are a source of various carbohydrates, including sugars, pectin substances, fiber, hemicelluloses, etc. [1,2,3,4].

The main digestible carbohydrates of *Schisandra chinensis* are glucose, fructose, and sucrose. They are classified as sugars, and their content in *Schisandra chinensis* is 3.8–9.5%. Glucose and fructose in fruits are always together with mineral salts, which is favorable for their rapid assimilation by the body. The total sugar content and their ratio is due to the taste of the Schisandra fruits.

The first studies of biologically active substances of *Schisandra chinensis* raw materials were carried out in the 1940s. In its juice, according to Yu.V. Branke, lemon (52%), apple (40%), amber (up to 4%), wine (up to 3%) and traces of oxalicum (0.22%) were found. The total acidity of *Schisandra chinensis* juice is up to 9.11% [5]. According to the results of studies carried out by FI Ibragimov and VS Ibragimova, the pulp of the fruit contains malic (27.4%), citric (69.9%) and tartaric acids [6].

According to the latest data, it is known that the fruit of *Schisandra chinensis* contains a complex of organic acids (27–32% in terms of dry matter), including 12% citric acid, 10% malic acid, as well as succinic and tartaric acids [7].

Organic acids are involved in the formation of taste, lowering the pH of the environment, stimulating the secretion of the pancreas and stimulating the activity of the intestines, enhancing its peristalsis. Therefore, eating *Schisandra chinensis*, rich in organic acids, contributes to the normal process of digestion.

In addition to non-volatile acids, small amounts of volatile acids are present in the composition of *Schisandra chinensis*: acetic, formic, valeric, nylon, etc. Acids of phenolic nature and their esters are contained in insignificant amounts. Volatile acids form the aroma of Schisandra fruit.

From trace elements, fruits contain Mn, Cu, Ni, Ti, Mo, Pb, Sn, and Zn, from macronutrients—K (53%), Mg (11–12%), Ca (7.9%), and P (6.7%). Ash is 1.15% dry matter [7]. According to the results of research by I.V. Krotova, in addition to the above microelements, fruits contain Cr, Al, Ba, and Sc, and ash, starch and sugars are 1.6%, 1% and 9.5%, respectively, for dried fruits [8].

Schisandra fruit is the main supplier of polyphenolic substances in the human diet. Polyphenols are characterized by versatility of action, which is due to the diversity of the structure of various groups of these compounds.

In accordance with the pathways of biosynthesis in plants, phenolic compounds are subdivided into eight groups: compounds of the C_6_ series, or simple phenols; compounds of the C_6_-C_1_ series, or phenolic acids (derivatives of benzoic acid); C_6_-C_2_ compounds, or phenolic alcohols and phenylacetic acids; compounds of the C_6_-C_3_ series, or hydroxycinnamic acids, phenylpropenes, and coumarins; compounds of the C_6_-C_4_ series, or flavonoids or isoflavonoids, as well as lignins and polymeric phenolic compounds—lignin, tannins, and melanins.

An important property of phenolic compounds is the ability to oxidize; they are especially easily oxidized in an alkaline environment. Phenols form brightly colored complexes with heavy metals. The antioxidant activity of many phenols is known and can be used as antioxidants.

The antioxidant activity of phenolic compounds is explained by the fact that they bind heavy metal ions into stable complexes, thereby depriving the latter of their catalytic action, and also serve as acceptors of free radicals formed during autoxidation (i.e., phenolic compounds are able to quench free radical processes) [9,10,11,12].

Phenolic compounds with two phenolic rings include: flavonoids, catechins, leukoanthocyanins, flavones, and anthocyanidins. Flavonoids differ in the degree of oxidation: the most reduced of them are catechins, the most oxidized are flavonols [9,10,11,12].

Many flavonoid compounds have a so-called P-vitamin effect on the human body. The number of P-active compounds reaches 150 forms. They have a variety of pharmacological effects; therefore, the scope of their therapeutic application is large. Bioflavonoids, in addition to normalizing and strengthening the state of capillaries and increasing their strength, have the ability to activate oxidative processes in tissues, as well as enhance the reduction of dehydroascorbic acid to highly active ascorbic acid. Thus, bioflavonoids increase the supply of vitamin C to the body. The total amount of P-active compounds in Schisandra fruits is 100 mg/100 g [13,14].

Catechins are the most studied group of flavonoids. Oxidation of catechins is especially intense in the presence of oxidative enzymes, which explains the rapid browning of freshly squeezed juice. They contain from 50 to 150 mg/100 g [13,14].

Leucoanthocyanidins are found in fruits together with catechins, but their content is higher than catechins. It is believed that it is the leucoanthocyanidins that are responsible for the unwanted discoloration of the fruit when cooked. Their content in Schisandra ranges from 30 to 190 mg/100 g. Flavones or flavonols, due to their high oxidation, have less effect on color change, and they are contained in a small amount (12.0–24.0 mg/100 g).

Anthocyanidin glycosides are called anthocyanins. Polyphenolic substances in anthocyanins impart coloring substances to fruits. Their content in Schisandra ranges from 20 to 95 mg/100 g. Anthocyanins change color when exposed to heat and cooling, as well as under the influence of biochemical processes.

Polyphenolic compounds significantly affect the quality of finished drinks. Violation of the integrity of fetal cells and the resulting darkening, as well as the development of oxidative processes during heating, are largely the result of a change in the chemical structure of polyphenolic compounds.

The astringency and astringent taste of *Schisandrachinensis* is due to tannins, which have an astringent taste, the ability to bind and precipitate complex protein substances, oxidation to red-brown compounds—flabofens, and the ability to bind to iron salts. The literature provides data on the content of tannins in Schisandra (0.11–0.33%) [15,16,17,18].

Tannins are complex amorphous high-molecular compounds, which include numerous phenolic hydroxides. 

When treated with dilute acids, hydrolyzable tannins decompose to form simpler phenolic and non-phenolic compounds.

Condensed tannins undergo further compaction in contrast to hydrolyzable substances when heated with dilute acids. By their chemical nature, they are polymers of catechins or leucoanthocyanidins or their copolymers [15,16,17,18].

Tannins are found mainly in the shell of the fruit, where they are mainly condensed. There are more of them in unripe fruits than in ripe ones [15,16,17,18].

In the production of drinks based on fruit and berry raw materials, the antiseptic properties of tannins, which are important for the storage of raw materials, are appreciated, as well as the ability to give drinks fullness and freshness of taste. Tannins contribute to the clarification of fruit and berry semi-finished products and drinks, due to their ability to precipitate protein substances.

Mineral substances play an important role in physiological processes in plant and animal organisms. Trace elements are necessary for humans in very small quantities, but their absence causes various diseases [15,16,17,18].

*Schisandra chinensis* fruits are the richest source of macro- and microelements. The fruits are rich in K, Na, Ca, and Mg, and give rise to alkaline compounds, regulating the acid–base balance.

G.N. Buzuk found the content of K, Ca, Mg and Fe (19.2; 0.7; 1.7 and 0.06 mg/g, respectively). According to the same author, Schisandra fruits are concentrators of Se and Ba [19].

The fruits also contain vitamins A, C, E, fumaric acid and stigmasterol [7]. Despite the insignificant content of vitamins in Schisandra fruits, even in small doses, they have significant activity and have a powerful effect on biological processes in organs and cells.

Schisandra seeds contain 38% fatty oils, the main components of which are linoleic acid (77%), linolenic acid (1.2%), and palmitic acid (1.4%) [7]. In addition, the presence of oleic, palmitoleic, myristic and stearic acids in fruits and seeds is noted [20].

In the 1950s, it was reported that up to 18.8% of substances extracted with methyl alcohol are present in unrefined fatty lemon oil. These include chlorophylls, sterols, rubberol alcohol, vitamin E, free acids, ether compounds and trace elements: Cu, Mn, Ni and Zn [21].

According to NI Suprunov, Schisandra seeds contain 28–31% fatty oil, which contains myristic, palmitic, stearic, oleic, linoleic, linolenic acids [22].

According to I. And Lapaev, up to 50.4% of oils were found in the seeds of schisandra, including 5.7% of essential oils [23]. According to DA Balandin, the yield of essential oils from seeds is from 1.6% to 1.9%, and from ripe whole berries—0.89% [24]. According to other sources, the content of essential oils in seeds ranges from 1.9 to 2.2% [15]. 

The main components of essential oils are monoterpenes: borneol, 1,8-cineole, citral, p-cymene, α- and β-pinenes. The essential oil also contains sesquiterpenes, such as ylangren, sesquicaren, hamigrenal, andα- and β-hamigrene [5,25].

Recently, both in Russia and abroad, studies have been carried out on the qualitative composition of Schisandra essential oil and the quantitative content of individual components [26]. For example, Chinese scientists have isolated 56 components of Schisandra seed essential oil by gas chromatography [8].

It is generally accepted that the main components providing the pharmacological action of Schisandra are dibenzo [a, c]—cycloocta-diene lignans, which are isolated from non-hydrolyzed fractions of seed oils. Their chemical structure, in contrast to other lignans, is based on an 8-membered carbon ring [7]. In particular, according to many studies, lignans are the main bioactive component of omija, and various functional activities, such as antioxidant, anti-cancer, and anti-inflammatory activity, of schisandrin, a representative substance among lignans, have been reported [27,28].

According to Japanese data, *Schisandra chinensis* fruits contain from 0.1 to 0.7% schisandrinA, pseudo-γ-schisandrin—0.3–0.5%, and schisandrolB—0.2–0.6% [27]. The schisandrincontent is 0.7–1.4% [28].

Schisandra fruits have a wide spectrum of pharmacological activity: they stimulate the central nervous system and the cardiovascular system, enhance the energy side of metabolism, and have anti-inflammatory, antioxidant, antimicrobial, antifungal, antitumor, and choleretic effects. The amount of Schisandra lignans has tonic and adaptogenic properties and stimulates the secretory activity of the stomach and the function of the gonads. The absence of side effects, cumulative properties and drug dependence is a valuable property of Schisandra preparations. 

Schisandra has a beneficial effect on carbohydrate metabolism by lowering blood sugar levels.

Research by V.A. Dorovskikh [29,30] found that cold exposure significantly reduces the resistance of experimental animals to fatigue, and especially in the first days of the experiment. Thus, a decrease in the resistance of experimental rats to dynamic and static load was revealed—a decrease in the duration of swimming of rats, as well as running on a treadmill and the time of “hovering” on a vertical grid.

In this regard, we set the task to determine the possibility of increasing the resistance of experimental animals to physical fatigue under conditions of inclusion in the diet of *Schisandra chinensis* extract.

## 2. Materials and Methods

### 2.1. Experimental Studies Conducted on Rats

The studies were performed in male albino rats. Charles River Wistar weighing from 220 to 250 g were obtained from the Institute of Cytology and Genetics of the Siberian Branch of the Russian Academy of Sciences (Novosibirsk, Russia). The rats were kept under standard conditions of temperature (22 ± 2 °C), humidity, and light (12 h light/dark cycles). The animals were fed rat food ad libitum and had free access to tap water. The rats were acclimated under these conditions for two days before the start of the experiment. The control and experimental groups of animals were randomized, 7–10 rats each. Laboratory animals lived in separate, ventilated cages, placed in a rack-shelf.

When working with animals, we were guided by the rules and international recommendations of the European Convention for the Protection of Animals used in experimental research. Group 1 served as control and received only food ad libitum, and they had free access to tap water. Group 2 was exposed to the cold, received only food ad libitum, and had free access to tap water. Group 3 was exposed to cold exposure, fed ad libitum, and received an aqueous extract of *Schisandra chinensis* in the amount of 100 mg/day. Group 4 was exposed to cold exposure, received food ad libitum, and received an aqueous extract of *Schisandra chinensis* in the amount of 150 mg/day. Group 5 was exposed to cold exposure, fed ad libitum, and received an aqueous extract of *Schisandra chinensis* in an amount of 300 mg/day. Methods for assessing physical performance were as follows. Every day, in the morning hours, the animals were placed for 3 h in a climate chamber of the Fentron Company of the German Democratic Republic at a temperature of minus 15 °C at 50% humidity for 28 days. In the operation of the chamber, air was provided to prevent oxygen hypoxia and a constant cooling mode was created.

Under these conditions, a persistent decrease in the temperature of the core of the body and pronounced biochemical changes occur, which are manifested in fluctuations in the content of peroxide products in accordance with the development of the stages of the adaptation process.

The main criterion for maximum performance (physical endurance) was the swimming time until complete exhaustion in water at a temperature of 40 °C for rats with a load equal to 10% of body weight. The weight—copper wire—was attached with a pin in the region of the sacrum. Signs of fatigue, with the manifestation of which the animals were removed from the pool, were: frequent and prolonged diving to the bottom, inability to hold the front limbs on the metal edging of the aquarium, prostration, “lateral position” after being removed from the water. The swimming technique is the most convenient for the integral characteristics of the state of the organism and its physical endurance, that only in life-threatening conditions can the animal be forced to “work until complete exhaustion”. Dynamic performance was also determined in the treadmill. The speed of the belt was 17 m/min, the angle of inclination was 100.

### 2.2. Plant Material and Extraction

The object of the study was an aqueous extract obtained from dried fruits of *Schisandra chinensis*, collected in the Amur region of the Russian Federation. The extraction efficiency is influenced by the temperature, the duration of the process and the type of extractant. Since at high temperatures biologically active substances of Schisandra can be inactivated, and the processes of polymerization of phenolic compounds with the formation of inactive forms, leading to turbidity, can be enhanced, the extraction temperature was chosen in the range of 40–50 °C. Since the polyphenolic substances that make up *Schisandra chinensis*, as well as vitamins and acids, are water-soluble compounds, water was used as a solvent.

The extracts were prepared taking into account the water absorption coefficient, which was determined experimentally. To prepare the extracts, we used crushed dry raw material in an amount of 500 g, passed through a sieve with a hole diameter of 2 mm, placed in an extractor and filled with distilled water in a 1:15 hydromodule. The extraction time was 6 h. The duration of pressing for six hours is due not only to the release of the maximum amount of biologically active components, but also to the “ripening” of the extract, which is expressed in the formation of fullness and harmony of taste. Extracts were analyzed using an Ultimate 3000 RS system (Dionex, Sunnyvale, CA, USA) in combination with an Amazon SL ion trap mass spectrometer (Bruker Daltonics, Bremen, Germany). Separations were performed on a Kinetex XB-C 18 column (150 mm × 2.1 mm × 1.7 μm, Torrance, CA, USA). The column was eluted with 0.1% formic acid in deionized water (A) and 0.1% formic acid in acetonitrile (B). A gradient program of 0 min—1% B, 60 min—26% B was used. The flow rate was 0.3 mL/min, and the column temperature was maintained at 25°C. A panel of 10 tasters worked to determine the organoleptic quality of the developed extracts. 

The studied samples of the extracts were detectable, color, taste and aroma and transparency. Each indicator of the quality of the extracts was assigned a score on a five-point scale. The maximum assessment of a sample of extracts for all organoleptic indicators was 20 points. The food and energy value of the samples was determined by the calculation method.

### 2.3. Chemical Composition

The bioactives were identified and quantified using the Agilent 1200. Method accuracy was expressed as percent recovery of the bioactives at low, medium and high concentrations in a range, each of which was analyzed in 10 samples.

### 2.4. Statistical Analysis

All data were presented as mean ± standard deviation (SD). The experimental design for all test groups was performed in such a way that first a general comparison between components was required, and then comparisons between pairs of components. Thus, differences between different groups were assessed using one-way analysis of variance (ANOVA) followed by a posteriori Bonferroni correction for multiple comparisons to identify pairs that matter. Single-tailed t-tests were used to test the hierarchy between mean values between groups. Differences in indicators were considered statistically significant at *p* < 0.05, *p* < 0.01, *p* < 0.001. All statistical analyses were performed using statistical software SPSS 10.0(SPSS Inc., Chicago, IL, USA).

## 3. Results

The content of biologically active substances in extracts of *Schisandra chinensis* (Turcz. Baill) are presented in Table 1.

As can be seen from the above data, the extracts obtained from the fruits of *Schisandra chinensis* are characterized by a high content of biologically active substances.

Organoleptic indicators of the quality of extracts from *Schisandra chinensis* are given in Table 2.

The nutritional and energy value of the obtained extracts is shown in Table 3.

As can be seen from Table 3, the obtained extracts have a high nutritional value and can be used as sources of biologically active substances in the production of functional drinks.

In scientific research to identify the pharmacological value of extracts, the method using physical activity is quite common. At the same time, it is assessed as the maximum duration of the load against the background of the course use of *Schisandra chinensis* extract for the formation of stable adaptation to muscle activity.

The animals received an extract from *Schisandra chinensis* against the background of systematically conducted muscle loads, which were modeled in accordance with the method proposed by Yu. P. Poholenchuk [29]. Repeated determination of the maximum running duration after 7, 14, 21, and 28 days of the experiment made it possible to establish that the developed extracts during the training cycle had a positive effect on the performance of animals in Table 4.

Thus, in experiments with determining the duration of running on the treadmill, a distinct act-protective effect was observed with the introduction of *Schisandra chinensis* extracts at a dose of 150 mg/day, against the background of reduced resistance to physical activity due to cold exposure.

The main criterion for maximum performance (physical endurance) was also the swimming time until complete exhaustion in water at a temperature of 40 °C for rats with a load equal to 10% of body weight. Signs of fatigue, with the manifestation of which the animals were removed from the pool, were: frequent and prolonged diving to the bottom, inability to hold the front limbs on the metal edging of the aquarium, prostration, and “lateral position” after being removed from the water. The swimming technique is the most convenient for the integral characteristics of the state of the organism and its physical endurance, that only in life-threatening conditions can the animal be forced to “work until complete exhaustion”. The results of the studies carried out are presented in Table 5.

It was found that exposure to cold significantly reduced the swimming resistance of rats on all days of the study. The introduction of *Schisandra chinensis* extract into the diet led to an increase in resistance to fatigue and an increase in the duration of swimming on all days of the experiment.

On the seventh day of the experiment, the swimming duration of intact rats was 145 ± 5.2 (min), in rats exposed to cold exposure—120 ± 6.2 (min) (*p* < 0.01), and with the introduction of *Schisandra chinensis* extract at a dose 150 mg/day for cold-adapting rats—240 ± 3.8 (min) (*p* < 0.01).

The introduction of *Schisandra chinensis* extract at a dose of 300 mg/day into the diet of animals increased the resistance of mice to fatigue compared to the control; the average swimming time of this group of rats was 200 ± 3.6 (min) (*p* < 0.01).

The introduction of *Schisandra chinensis* extract into the diet at a dose of 100 mg/day increased the swimming duration of rats insignificantly and amounted to 170 ± 5.4 (min). A similar pattern persisted until the 28th day of observation.

## 4. Discussion

The processes of human interaction with the environment constitutes one of the most important problems of modern biomedical science. The adaptation of the human body to cold climatic conditions has been devoted to a large number of studies [31,32,33].

Cold exposure causes a violation of oxidative phosphorylation, disrupts the function of organs, and reduces their working capacity and resistance to physical activity. At the same time, the oxidative metabolism of muscles changes, the efficiency of muscle contraction decreases, the oxidative phosphorylation in mitochondria is uncoupled and the synthesis of ATP is disrupted [33,34,35].

During cold exposure, the body manages heat conservation through well-known channels but also by specialized thermogenic functions such as metabolism in brown adipose tissue [36]. It seems that especially simple cognitive tasks are affected by the cold, while on more complex tasks, performance may even improve in mild to moderate cold [37]. Specialized studies show that repeated exposure to severe cold on the whole body of individual volunteers, mainly Caucasians, leads to a decrease in the sensation of cold, but does not cause serious physiological changes [38]. Effects of cold adaptation and high-fat diet on the metabolic responses as well as cold resistance to acute cold exposure were investigated in rats with emphasis on elucidating the mechanism underlying the favorable effect of high-fat diet in a cold environment previously reported.

An increment in body weight was greater in rats on a high-fat diet and smaller in cold-adapted rats than in control rats. However, food intake was significantly greater in cold-adapted rats [39].

On exposure to cold, an increase in body weight would be adaptive. Some wild mammals increase their weight and body fat in winter, but adult laboratory mice, after transfer from 21 to −3 °C, lose weight, largely owing to loss of fat. In this they resemble laboratory rats transferred to a cold environment. Similarly, inbred mice reared at −3 °C are usually lighter, at all ages, than controls at 21 °C [40].

The study by Kashimura O. showed that the development of a higher metabolic rate during acute hypothermia in trained rats is associated with an increased level of norepinephrine and adrenaline-independent thermogenesis without shivering, as well as an increased metabolic rate at rest at an ambient temperature of 25 °C [41].

*Schisandra chinensis* is known as a plant adaptogen with many beneficial biological properties.

*Schisandra chinensis* and extracts obtained from it increase the nonspecific reactivity of the body, stimulate the hypothalamo-pituitary-adrenal system, and increase the activity of antioxidant defense mechanisms. They stabilize biological membranes, protect them from decay during overload, and promote the processes of synthesis and metabolism, a kind of renewal, rejuvenation of the body.

Thus, in experiments with determining the duration of running on the treadmill, a distinct act-protective effect was observed with the introduction of *Schisandra chinensis* extracts at a dose of 150 mg/day, against the background of reduced resistance to physical activity due to cold exposure.

The main criterion for maximum performance (physical endurance) was also the swimming time until complete exhaustion in water at a temperature of 40 °C for rats with a load equal to 10% of body weight. Signs of fatigue, with the manifestation of which the animals were removed from the pool, were: frequent and prolonged diving to the bottom, inability to hold the front limbs on the metal edging of the aquarium, prostration, and “lateral position” after being removed from the water. On the seventh day of the experiment, the swimming duration of intact rats was 145 ± 5.2 (min), in rats exposed to cold exposure—120 ± 6.2 (min) (*p* < 0.01), and with the introduction of *Schisandra chinensis* extract at a dose 150 mg/day for cold-adapting rats—240 ± 3.8 (min) (*p* < 0.01).

The introduction of *Schisandra chinensis* extract at a dose of 300 mg/day into the diet of animals increased the resistance of rats to fatigue compared to the control; the average swimming time of this group of rats was 200 ± 3.6 (min) (*p* < 0.01).

The introduction of *Schisandra chinensis* extract into the diet at a dose of 100 mg/day increased the swimming duration of rats insignificantly and amounted to 170 ± 5.4 (min). A similar pattern persists until the 28th day of observation. The intake of *Schisandrachinensis* is not associated with serious side effects [42,43,44,45,46]. The beneficial effect of *Schisandra chinensis* on physical activity would be associated with directed energy production and distribution, as well as with the fighting of fatigue-induced oxidative stress [47,48,49].

For a better exploration of the benefits and future applications of the *Schisandrachinensis* extract, more in vivo studies and multicenter randomized double-blind studies should be performed.

## 5. Conclusions

Thus, the analysis of the presented experimental data indicates that the developed extracts in the amount of 150 mg/day equally increase the swimming duration of rats adapting to the cold on all days of the experiment.

The results of this series of experiments showed a gradually increasing effect of increasing the physical performance of rats with prolonged (28 days) administration of the developed drinks, which coincides with the literature data regarding a number of other adaptogens and indicates the presence of cumulative properties of biologically active substances of *Schisandra chinensis* extract [47,48,49].

These data served as the basis for determining the most acceptable and effective dose of the extract prepared by us for further research in order to determine its adaptogenicproperties. This dose is 150 mg/day.

## Figures and Tables

**Table 1:** The content of biologically active substances in extracts from *Schisandra Chinensis* (Turcz. Baill).

Raw Materials	BAS Content,%			
	Tannins	Phenolic Compounds	Anthocyanins	Organic Acids
*Schisandra Chinensis* (Turcz. Baill).	0.085 ± 0.001	0.025 ± 0.002	0.0072 ± 0.001	2.18 ± 0.05

**Table 2:** Organoleptic evaluation of extracts based on *Schisandra Chinensis* (Turcz. Baill).

Indicator Name	Significance Factor	Values, Points
Color, transparency	0.3	1.5
Scent	0.2	1.0
Taste	0.3	0.9
General impression	0.3	1.5
Complex indicator	1.0	4.9

**Table 3:** Nutritional and energy value of *Schisandra Chinensis* (Turcz. Baill) (100 g).

Indicator Name	Value
Mass concentration of proteins, g	2.6
Mass concentration of carbohydrates, g	25.3
Mass concentration of organic acids, g	25.7
Energy value, kcal	170

**Table 4:** Duration of running on the treadmill of rats under cold stress against the background of the introduction of *Schisandra chinensis* extract (in minutes).

Animal Groups	7 Day	14 Day	21 Day	28 Day
Intact	10.2 ± 1.6	12.0 ± 0.53	1100 ± 0.38	11.7 ± 0.83
Exposed to the cold impact	x 7.8 ± 0.4	x 9.2 ± 0.52	x 9.5 ± 4.7	x 8.8 ± 0.46
Cold + *Schisandra chinensis* extract 100 mg/day	xx 11.0 ± 2.1	xx 12.4 ± 2.2	xx 12.8 ± 1.4	xx 13.1 ± 4.4
Cold + *Schisandra chinensis* extract 150 mg/day	xx 13.6 ± 2.4	xx 17.8 ± 4.2	xx 18.4 ± 2.1	xx 17.0 ± 4.1
Cold + *Schisandra chinensis* extract 300 mg/day	xx 12.8 ± 1.6	xx 13.0 ± 5.17	xx 15.0 ± 3.25	xx 14.2 ± 1.8

Note: x and xx are values that significantly differ from the values of intact x and xx groups of animals exposed to cold effects, *p* < 0.05 *n* = 30.

**Table 5:** Duration of swimming of rats with prolonged cold stress and against the background of the use of *Schisandra chinensis* extract (in minutes).

Animal Groups	7 Day	14 Day	21 Day	28 Day
Intact	145 ± 5.2	152 ± 3.4	159 ± 4.3	162 ± 3.2
Exposed to the cold impact	x 120 ± 6.2	x 124 ± 2.1	x 135 ± 3.0	x 158 ± 3.3
Cold + *Schisandra chinensis* extract 100 mg/day	xx 170 ± 5.4	xx 174 ± 4.2	xx 181 ± 2.4	xx 185 ± 3.4
Cold + *Schisandra chinensis* extract 150 mg/day	x 240 ± 3.8	xx 250 ± 4.2	xx 258 ± 3.5	xx 290 ± 5.1
Cold + *Schisandra chinensis* extract 300 mg/day	x 200 ± 3.6	xx 210 ± 3.3	xx 214 ± 4.5	xx 225 ± 7.8

Note: x and xx are values that significantly differ from the values of intact x and cold-exposed xx groups of animals *p* < 0.01 *n* = 30.

## Data Availability

The data presented in this study are available on request from the corresponding author.
